# A strangulated internal hernia beneath the left external iliac artery after radical hysterectomy with laparoscopic pelvic lymphadenectomy: a case report and literature review

**DOI:** 10.1186/s12893-021-01249-5

**Published:** 2021-05-31

**Authors:** Zhenxing Zhang, Gengyuan Hu, Minfeng Ye, Yu Zhang, Feng Tao

**Affiliations:** Department of Gastrointestinal Surgery, Shaoxing People’s Hospital (Shaoxing Hospital of Zhejiang University), Shaoxing, 312000 China

**Keywords:** Hernia, Intestinal obstruction, Iliac artery, Lymphadenectomy, Case report

## Abstract

**Background:**

Ileum obstruction due to internal hernia beneath external iliac artery after pelvic lymph node dissection (PLND) is extremely rare. We reported a case of acute strangulated internal hernia between the left external iliac artery and psoas major as late complication of laparoscopic hysterectomy with pelvic lymphadenectomy.

**Case presentation:**

A 46-year-old woman, who with histories of laparoscopic hysterectomy, bilateral salpingo-oophorectomy and PLND 9 years ago for the cervical malignant tumor, open appendectomy 18 years ago, visited our hospital complaining of aggravated left lower abdominal pain, bloating, nausea and vomiting from few hours ago. Left abdomen distention, tympanitic with rebound tenderness and muscular tension was detected during physical examinations. Accompanying with elevated inflammatory markers and mild intestinal dilatation showed in lab results and contrast-enhanced computed tomography (CT) respectively. After carefully reading the CT images, a small bowel was found between the left external iliac artery (EIA) and the psoas major, combined with the patient's surgical history, we suspected it might be internal hernia. Eventually, the emergency laparoscopic laparotomy confirmed our conjecture, the gap between the iliac vessels and the psoas major was closed with an absorbable suture, the patient was discharged on the fourth postoperative day.

**Conclusion:**

Primary closure of peritoneal fissue maybe an effective measure to potentially prevent internal hernia. The choice of surgical approach for pelvic tumors still needs further exploration but faster diagnosis and immediate laparotomy might promise a better prognosis.

## Background

Pelvic lymphadenectomy is a standard procedure for cervical, ovarian, bladder and prostate cancer to improve the R0 resection. There have been few documented cases of internal hernia as late complication of laparoscopic hysterectomy with PLND. There have been several pelvic hernia cases in recent years reported that small bowel beneath the external iliac vessel, common iliac vessel or the obturator nerve. Internal herniation of the ileum beneath the external iliac artery after pelvic lymphadenectomy is extremely rare all over the world that has only been reported in six cases in previous English-language literature (Table 1). In general, acute acquired internal hernia is admitted to hospital with acute abdomen, and the most patients will undergo an emergency surgery even bowel resection [1]. Thus, rapid and accurate diagnosis of bowel hernia is crucial for immediate surgery before necrosis occurs, which is challenged to be roundly implemented in clinical practice [2].

**Table 1:** Reported cases of internal hernia beneath the EIA after PLND in English-language literature

Patient	Diagnosis	Operation	Operative approach	Hernial orifice	Latency	Bowel resection	Repair method	Treatment approach	Year	Countries or regions	Citation
67 years, female	Cervical cancer	Extended hysterectomy and PLND	Laparoscopic	Right EIA	3 mo	Yes	With peritoneum	Open	2008	Korea	Kim et al
56 years, female	Ovarian cancer	Total abdominal hysterectomy, omentectomy, appendectomy, radical retroperitoneal LND	Open	Left EIA	4 y	No	Unrepaired	Laparoscopic	2013	Norway	Dumont et al
50 years, male	Prostate cancer	Radical prostatectomy and PLND	Robot-assisted	Left EIA	1 y	Yes	Arteriotomy and repair with collagen patch and peritoneum	Open	2016	Switzerland	ViktorinBaier et al
64 years, male	Prostate cancer	Radical prostatectomy and PLND	Robot-assisted	Right EIA	1 y	Yes	Unrepaired	Open	2018	USA	Kambiz et al
72 years, male	Prostate cancer	Radical prostatectomy and PLND	Robot-assisted	Left EIA	2 mo	Yes	Unrepaired	Open	2019	Japan	Ninomiya et al
68 years, female	Endometrial adenocarcinoma	Radical hysterectomy and PLND	Laparoscopic	Between right EIA and EIV	7 y	Yes	With peritoneum	Open	2020	Germany	Felix et al
56 years, female	Cervical cancer	Radical hysterectomy and PLND	Laparoscopic	Left EIA	9 y	No	With peritoneum	Open	2020	China	Our case

## Case presentation

**Fig. 1:**
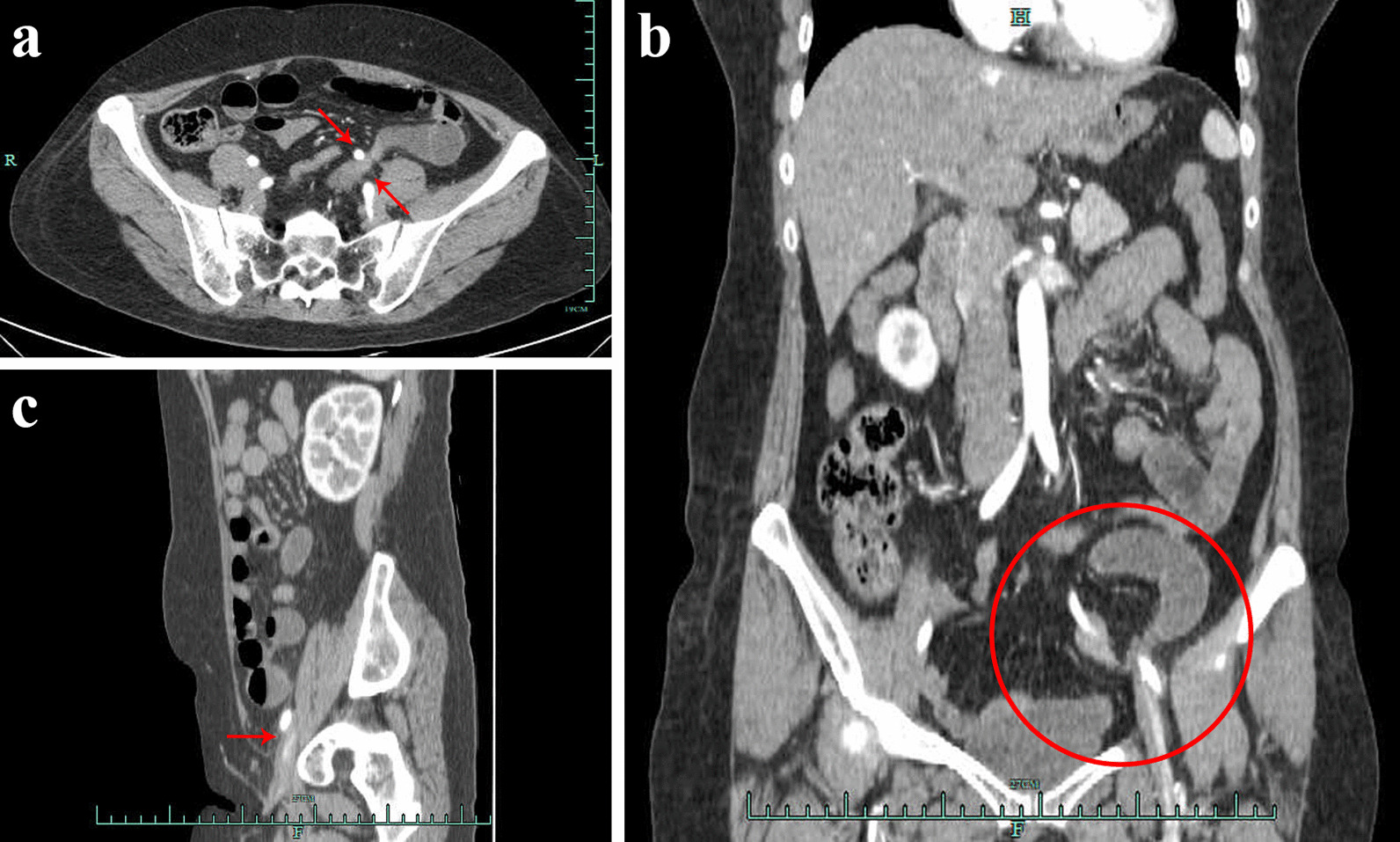
The preoperative contrast-enhanced CT scan. **a** axial view showing herniation of small bowel with abrupt reduction of caliber (red arrows) between left EIA and psoas major. **b** coronal view showing the site of herniation and distension of proximal bowel loop (red circle). **c** sagittal view showing herniation of small bowel (red arrow) beneath the left EIA

**Fig. 2:**
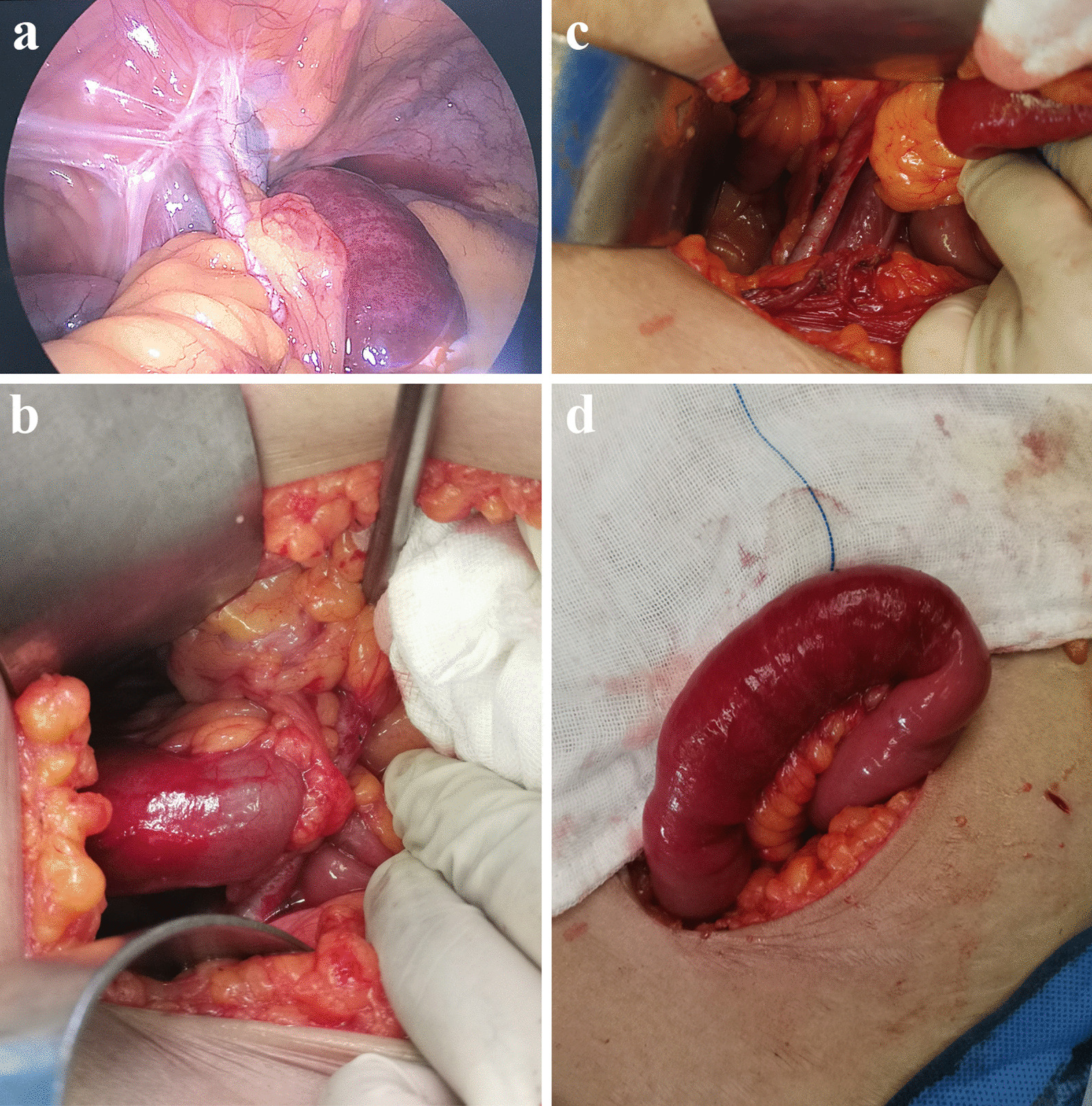
Intraoperative sight. **a**, **b**, about 10 cm ileum, ischemic, in purple, herniated into the gap between the left EIA and psoas major in the laparoscopic and open visual field. **c** the small bowel was reduced from the gap between the left EIA artery and psoas major. **d** the purple ischaemic bowel back peristalsis and resumed to pink

A 46-year-old woman visited our hospital complaining of aggravated left lower abdominal pain, bloating, nausea and vomiting for few hours. 9 years prior to presentation, the patient underwent a radical trachelectomy with laparoscopic pelvic lymphadenectomy for the cervical malignant tumor. In addition, the patient underwent an open appendectomy 18 years ago. A physical examination revealed an acute localized peritonitis with lower left abdominal tenderness, rebound tenderness, muscle tension locally. Laboratory tests showed slightly elevated inflammatory markers (CRP: 0.88 mg/l, WBC: 10.59*10^9^/L, NE: 82.5%). The uterus and accessories ultrasound and lower abdominal CT scan showed no other abnormalities besides hysterectomy. Considering the patient's symptoms and abdominal signs, we further performed the whole belly contrast-enhanced abdominal CT which suggested slight intestinal dilatation and a small amount of pelvic fluid. After careful reading of the CT images, a small bowel was found between the left EIA and the psoas major (Fig. 1), combined with the patient's surgical history, we suspected it might be an acute acquired internal hernia. Then we performed a laparoscopic laparotomy for the suspected ileus from the fissure. Intraoperative findings: about 10 cm ileum, ischemic, in purple, 2 m back from the cecum herniated into the gap between the EIA and psoas major (Fig. 2a, b). The dilated ileal wall was edematous and fragile, which could not be effectively clamped under laparoscopy, then we converted to open surgery. Carefully released the adhesion of the hernia orifice, removed the incarcerated loop from the hernial orifice, the hernial orifice consisted of the left EIA and the psoas major appeared (Fig. 2c). After warming the purple bowel with hot salt water towel, the purple bowel back peristalsis and resumed to pink (Fig. 2d), so we didn't remove any part of the intestine. A running suture with an absorbable 3–0 suture was used for primary closure of the sheath of left EIA and parietal peritoneum to prevent further re-herniation. The gastrointestinal decompression tube was removed on the first post-operative day after the patient had anal exhaust, and the patient was discharged on the fourth postoperative day.

## Discussion and conclusion

Comparing with adhesion, internal hernias is a rather rare cause of small bowel obstruction after surgery, which holds 5.8% accountability. More than 90% of all internal hernias are caused by natural or artificial orifices built by the intestine [3]. An internal hernia caused by a vascular structure, is certainly an extremely rare form of hernia. To our best knowledge, the case above is the first case in China, even rare throughout the world.

Our patient was firstly diagnosed as small bowel obstruction according to the physical examination, while strangulated internal hernia beneath the left EIA was confirmed with abdominal enhanced CT preoperatively. Previously literatrues reported seven of nine cases of internal hernia caused by vessels after pelvic lymphadenectomy underwent bowel resection to remove the ischemic segment [4]. To reduce the risk of bowel ischemia, rapid diagnosis and emergent surgery have been identified as a major contributing factor for treating patients with internal hernia after pelvic lymphadenectomy. Generally, internal hernia of the small intestine is difficult to make a diagnosis. However, the diagnosis of internal hernia beneath the EIA by a preoperative contrast-enhanced CT scan helped to detect the correlation between small bowel and vessels [5]. In the present case, contrast-enhanced CT also provided important auxiliary information for operation in time to avoid bowel resection.

Several options of treatment for internal hernia beneath the EIA have been described in the previous reports to prevent its recurrence. Kim et al. [6]closed the orifice with the peritoneum. Similarly, Felix et al. [4] also grafted the peritoneum to close the orifice. Another case has been reported by Viktorin-Baier et al. [7] that an arteriotomy of 2 cm and end-to-end anastomosis of the elongated artery was performed including repairing with collagen patch and peritoneum. In contrast to Kim and Felix, Dumont et al.[8] and Ninomiya et al. [9] did not close the orifice in order to avoid the injury of the iliac artery. Another option was to close the orifice with a permanent mesh [10], but no case has been reported by previous English-language literatures to our knowledge. As a result, we reduced the herniated bowel, and decided to repaired the orifice with the peritoneum to prevent the recurrence of a herniation. Due to a lack of experience with permanent mesh directly on an artery, we did not perform this procedure also for minimizing the risk of infection.

PLND including skeletonization of iliac vessels has been accepted as a standard procedure for patients with cervical [11], ovarian [12] and urogenital cancer [13, 14]. Once the lymphatic tissue was removed, a potential hernial defect was frequently created between the arching retroperitoneal vessels. In spite of logical advantages, preventive reperitonealization after pelvic lymphadenectomy has not been shown to change outcomes in postoperative morbidity. In contrast, this internal hernia following retroperitoneal lymphadenectomy may be due to tortuous, elongated iliac arteries in special patients.

Pelvic vasculature skeletonization can cause fissure formation, which may subsequently lead to pelvic internal hernia. Primary closure of peritoneal fissue maybe an effective approach to prevent the initiation of hernia in the first place. It is still disputable whether opening the belly or applying laparoscopy is the better choice to remove pelvic tumor, and further investigation is warranted. Moreover, internal hernia is a powerful " killer " of acute abdomen, faster diagnosis and immediate laparotomy might promise a better prognosis.

## Data Availability

All patient data and clinical images adopted are contained in the medical files of Shaoxing People’s Hospital. The data supporting the conclusions of this article are included within the article and its figures and tables.

## Abbreviations

PLND: Pelvic lymph node dissection
CT: Computed tomography
EIA: External iliac artery
CRP: C-reactive protein
WBC: White Blood Cells
NE: Neutrophils
